# Spectators lead to overconfidence and risk-taking in males in a motor task

**DOI:** 10.1038/s41598-025-18048-0

**Published:** 2025-09-12

**Authors:** Fabian Pelzer, Kai Leisge, Christian Kaczmarek, Sabine Schaefer

**Affiliations:** 1 Present Address: Institute of Sport Sciences, Saarland University, Saarbrücken, Germany; 2 German University for Prevention and Health Management, Saarbruecken, Germany

**Keywords:** Spectator effects, Performance prediction, Risk-taking, Gender, Motor task, Neuroscience, Psychology

## Abstract

We often perform motor tasks in front of other people, and this may help or hinder our performances. Previous research mainly focused on changes in motor performance, and less is known about spectator influences on performance predictions. An accurate performance prediction in motor tasks is important, as it supports optimal performance. The present study examined whether being observed by spectators influences not only performance, but also performance predictions in a motor task. We tested 341 participants on a speeded cup-stacking task in a within-subjects design: co-acting (performing concurrently with others) versus performing alone in front of an audience. In half of the trials, participants predicted how many cups they would be able to stack in the upcoming trial. Overconfidence, by predicting performances that were too high, resulted in failed trials. Being watched by others led to motor performance decrements. Both males and females left a safety margin in their performance predictions in the co-acting condition. Being watched by others led males to increase their overconfidence and failure rate, whereas females left a safety margin in their predictions, maintaining a stable failure rate in front of spectators. Our results indicate that spectators not only influence motor performance, but also performance predictions.

**Supplementary Information:**

The online version contains supplementary material available at 10.1038/s41598-025-18048-0.

## Introduction

In daily life, solving tasks often takes place in the presence of others (e.g., in a professional or sports context). Spectators may be uninvolved and passive, co-acting, or merely observing the actor. In general, the performance of simple or well-learned tasks often benefits from the presence of spectators, a phenomenon referred to as “social facilitation”, while the performance of complex or not well-learned tasks tends to deteriorate (“social inhibition”)^1^. Effects of spectators on performance have been shown for both cognitive and motor tasks (^1^, for a recent review, see ^2^). Early empirical approaches often kept the spectator condition as pure as possible. For example, researchers implemented a “mere presence” condition in which spectators did not express any interest in the performance of the actor or were even blindfolded^3^. However, in real-life situations, spectators often evaluate a person’s performance, leading to more pronounced spectator effects that may be moderated by additional factors such as personality traits, performance level, or previous experiences^4^.

The context of sports is a real-life scenario, in which (recreational) athletes are often observed by others. Athletes may be briefly observed (and potentially judged or admired) during a morning run (e.g., running uphill at a low speed), an evening gym workout (e.g., successfully performing a challenging set of repetitions), or most obviously in professional sports, where spectators pay to watch athletes perform. According to Strauss (2002), being watched by others enhances performance in condition-based motor tasks (like running or weight-lifting), but impairs performance in coordination-based tasks (like juggling or gymnastics)^1^. In cases where a good performance is highly important, such as a free throw in the last minute of a basketball game, spectator effects may lead to a significant performance deterioration, consistent with the phenomenon of choking under pressure^5^.

In motor and sports-related tasks, an adequate perception of one’s abilities not only helps to achieve maximum performance, but also prevents excessive risk-taking, which can lead to reduced performance or even injuries. For instance, this includes strategies for managing effort in endurance sports events (pacing strategies) or setting a target weight for the next attempt in weightlifting competitions^6–9^.

However, self-assessment is not uniform across individuals, as factors such as gender influence how abilities are perceived. In various cognitive tasks, men have been shown to overestimate their abilities more strongly and to exhibit higher self-esteem than women^10–14^. More recently, this effect has also been observed in motor tasks. Obling et al. (2015) found that men tended to overestimate their physical fitness levels more strongly than women when comparing self-reported physical fitness with objectively measured cardiovascular fitness (maximal oxygen consumption)^15^. In a more specific sports context such as endurance sports, men show an overestimation in their ex-ante performance prediction of their marathon finish times^9,16^. This leads to over-pacing, resulting in a more pronounced slowdown in the later stages of the race, which is associated with a greater performance deterioration compared to a balanced pacing strategy^7,9^. In recreational skiing, where overestimation of performance entails a direct risk of injury, Luppino et al. (2023) found that those who overestimated their skiing performance level were predominantly physically unprepared males^17^. In their recent study, Bühren et al. (2024) found choking under pressure in alpine skiing to be particularly pronounced among men. The authors argue that the most likely explanations for their results are gender differences in response to expectations and the tendency to choke under pressure^18^.

Differences in self-assessment of abilities can also be moderated by age, as children and older adults show a stronger tendency to overestimate their performance compared to young adults^16,19–23^. An accurate self-assessment of motor performance is especially crucial for older adults to prevent falls or collisions with obstacles. For example, Kawasaki and Tozawa (2020) found that older adults overestimate their motor abilities (estimating the distance they could cover by taking two maximum-effort steps forward), which can increase the risk of falls and accidents. Their results suggest that this overestimation is linked to age-related declines in motor abilities^20^.

In general, there is limited evidence on how spectators influence self-assessments of abilities. While previous studies have shown that the presence of peers can increase risk-taking behavior, particularly among male adolescents^24,25^, these findings primarily relate to social dynamics within peer groups, in which conformity and social bonding mechanisms drive behavior, rather than to the mere presence of spectators^26^. However, they suggest that being observed by others, whether peers or passive spectators, may influence self-assessment and decision-making.

We assume that self-assessment of performance and strategic decision-making are influenced by spectators (e.g., leading to more overestimation of one’s own performance and risk-taking). These influences may be moderated by gender or age.

A research paradigm developed by Riediger et al. (2006) proposes to systematically test performance levels to assess the maximum-manageable task-difficulty for each individual^27^. The difference between the chosen task difficulty and the maximum-manageable task-difficulty is the “selection margin”. People can overestimate their performance and therefore be overconfident (i.e., choosing task difficulty-levels that are too high, “progressive” selection margins), neutral, or underestimate their performance and therefore be underconfident (choosing difficulty-levels that are too low, “conservative” selection margins). Studies using this paradigm for different motor or cognitive tasks have detected age differences in selection margins, with children and teenagers showing a higher tendency to overestimate their performance compared to young adults^19,28^, while over- or underconfidence in older adults seems to be influenced by the physical risk of the motor task^22^. Gender differences were also found, with males showing higher levels of overconfidence than females^19^.

To the best of our knowledge, no previous study has examined how spectators affect individuals’ performance predictions of motor task performance. For the current study, we used a speeded task, repeatedly stacking cups to construct and deconstruct little pyramids. Participants stacked concurrently with the others (co-acting), as well as alone in front of all other participants watching them. In addition, for half of the trials, participants predicted how many cups they would be able to stack in the upcoming trial. They received points corresponding to their predicted score if – and only if – they managed to stack at least the predicted number. If they failed to reach the predicted score, they did not receive any points for this trial. The paradigm therefore punished overestimations.

We predicted that performances in the stacking task would suffer from the presence of spectators, since stacking requires predominantly coordination^1,2^. Gender can influence spectator effects^29^ and is included in the analyses. In addition, we assumed that spectators would also influence performance predictions, with males showing higher overestimations of their performance compared to females, especially in front of an audience.

## Methods

### Participants

We conducted a power analysis using the G-Power 3 software^30^ to estimate the required sample sizes. The first analysis focused on the effect of spectator-induced performance reductions on stacking. The review by Strauss (2002) and the recent meta-analysis by van Meurs et al. (2024) show that spectator effects on motor performance tend to be small^1,2^. A power analysis for a paired sample t-test with a significance level of 0.05 and a power of 0.95 indicated that a sample size of 175 would be sufficient to detect a small effect (dz = 0.25) for the effect of spectators on stacking performance.

To the best of our knowledge, no previous study has examined how spectators affect individuals’ self-assessment of task performance. However, previous studies using the selection margin paradigm have revealed age and gender differences in selection margins with medium effect sizes^19,22^. For spectator and gender influences on selection margins, we conducted a power analysis (ANOVA: repeated measures, within-between interaction) with a significance level of 0.05, a correlation of the repeated measures (2: spectator, co-action) of *r* = .50, and a power of 0.95. The analysis indicated that a small effect size of *f* = 0.10 would require a total sample of 328 participants.

We assume that the sample size of more than 300 participants will be sufficiently large to not only detect main effects of age and gender on selection margins, but also potential interactions of spectators, age and gender on selection margin decisions.

We tested 341 (Caucasian) participants in a within-subjects design. The sample’s gender distribution is balanced, with 183 males aged from 8 to 89 years (Age: *M* = 28.3, *SD* = 18.6) and 158 females within the ages of 8 to 93 years (Age: *M* = 27.8, *SD* = 19.8). Participants were asked to indicate their gender with the question: “You are: male/female?”. None of the participants of the current study identified themselves as non-binary concerning gender. Participants were recruited in contexts in which they met in group settings in their everyday lives, for example in exercise classes, university seminars, choirs, leisure time activity groups or sports clubs. They were asked to participate in the testing session in groups of 4 to 6, and did not receive financial reimbursement for their participation. The university students received course credit for their participation. Exclusion criteria were motor or health impairments, like acute injuries of the upper extremities. No other specific inclusion or exclusion criteria were defined prior to the study. All eligible participants were included in the data analyses. The study was approved by the ethics committee of Saarland University (“Ethikkommission der Fakultät für Empirische Humanwissenschaften und Wirtschaftswissenschaft”, Ethics Application 17 − 08). All participants provided written informed consent prior to participation, in accordance with the Declaration of Helsinki. For minors, written informed consent was obtained from a parent or legal guardian.

### Apparatus and experimental task

Participants performed the motor task cup-stacking (“stacking”) in a within-subjects design. Speeded cup-stacking is a 3-dimensional skill that requires multi-joint coordination. Stacking consists of the repeated construction of 4-3-2-1 pyramids with cups (height: 4 cm; diameter: 3 cm) on a green carpet-like surface (30 × 20 cm) in a seated position. A fully constructed pyramid resulted in a score of 10 (since 10 cups are used to build it). After a pyramid was built, it had to be fully deconstructed (including the base) before the next pyramid could be started. If the pyramid collapsed before it was fully constructed, this attempt did not contribute to the overall score of the trial. Participants had two spare cups at their disposal, which they could use when a cup dropped to the floor during a trial. Each trial lasted for 30 s. Participants were instructed to construct and deconstruct their pyramids as quickly as possible throughout the trial, and to keep a running count of their score during each trial. The dependent variable was the number of stacked cups during a trial (e.g., two full constructions of the pyramid – 10 cups each – and 4 successfully stacked cups in the third attempt results in a score of 24).

Stacking was performed individually (co-acting simultaneously with the other participants), or while being watched by the other participants (spectator conditions). In the co-acting condition, participants were seated next to each other at separate tables in the same room with the experimenter and the other participants. Each participant stacked individually, with all trials starting simultaneously. This simultaneous execution of the task prevented systematic observation of other participants in the co-acting condition. In the spectator conditions, participants walked to a separate workplace and stacked in front of the others. Note that the other participants were in the spectator role in this situation. Spectators were instructed to remain silent and to observe the performances, without “cheering”, “booing”, or interacting with the performer. An experimenter was present in the room during all conditions.

Additionally, in half of the trials, participants were asked to predict their performance for the trial ahead. Participants were instructed that they would receive the predicted number as “points” if - and only if - they managed to stack at least their predicted score (e.g., predicting 28 cups, stacking 30 cups results in receiving 28 points; predicting 28 cups, stacking 27 cups results in 0 points). The paradigm therefore “punished” overestimations of one’s performance, because participants did not receive any points for unsuccessful trials (zero-point trials). To support accurate predictions, participants were instructed to continue stacking for the full trial duration, even after reaching their predicted goal, in order to assess their maximum performance for each trial.

Based on the selection margins paradigm^27^, we calculated the difference between the predicted and the actually achieved performance for each performance-prediction trial (selection margin score, SMS), resulting in either overestimation (values larger than 0), underestimation (values smaller than 0) or exact prediction of performance (value equal to 0) for every trial.

### Procedure

Participants were tested with four to six persons per group. Of the 341 participants tested, 239 (70%) were tested in gender-homogeneous groups, and 102 (30%) in gender-heterogeneous groups. Each session lasted ~ 90 min. Each participant attended one session. After the assessment of demographic information, the stacking task was explained. Initially, participants were given two minutes to familiarize themselves with the cups and the task.

Participants were instructed to perform at their best on each trial. The study was framed as an investigation of spectator effects on performance. Gender differences were not emphasized at all. Participants stacked in a co-acting situation for 10 trials (stacking pretest). After that, the four experimental conditions were assessed: co-acting without performance prediction (condition 1; eight trials), co-acting with performance prediction (condition 2; eight trials), stacking in front of spectators without performance prediction (condition 3; five trials), stacking in front of spectators with performance prediction (condition 4; five trials). The reason for fewer trials in front of spectators compared to the co-acting condition was that the spectator blocks took considerably longer. Participants had to watch each other perform the task, and we wanted to avoid fatigue and boredom. Breaks of approximately two minutes were provided between the different conditions to prevent fatigue and maintain concentration. When stacking in front of spectators, the order of participants was randomized. When performance predictions had to be made, participants verbalized their prediction before each trial. Note that the groups were equally distributed across four different counterbalancing orders of the four experimental conditions to control for the influence of practice. At the end of the testing session, participants performed eight trials of co-acting stacking (posttest).

### Statistics

The statistical analyses were conducted using R Statistical Software for Windows (version 4.2.1)^31^. Two different linear mixed-effects models were used, with participant as a random effect, and age, spectators (2; spectators, no spectators), performance prediction (2; prediction, no prediction), and gender (2; male, female) as fixed effects to predict the influence on either stacking performance or SMS. The mixed-effects models were conducted with the nlme R package^32^. The model predictors were checked for linearity, but no violations were found. Descriptive statistics were calculated via the psych R package^33^.

In addition, a repeated-measures ANOVA with the within-subjects factor spectator (2; spectator, no spectator) and the between-subject factor gender (2; male, female) was calculated to compare the proportion of males and females scoring zero points in the spectator and co-action condition. A post-hoc independent t-test was carried out to compare the proportion of males and females scoring zero points (zero point trials) in the spectator condition due to overestimation.

We calculated two additional repeated-measures ANOVAs on selection margin scores with the within-subjects factor trial (8 trials for the co-acting condition; 5 trials for the spectator condition) and the between-subject factor gender (2; male, female). Note that due to missing values, 301 participants were included in this additional analysis for the co-action condition. For all analyses, the alpha level was set to 0.05.

Data and the analysis code can be accessed here: https://osf.io/bvc3s/?view_only=1a835e6a602b49cc9c3c2d59d15327a7.

## Results

### Main hypothesis: changes in stacking performance by condition

Reliability coefficients were calculated for all 44 stacking trials of the respective study (10 trials pretest, 16 co-acting trials, 10 trials in front of spectators, 8 trials posttest). Reliabilities were excellent, Cronbach’s Alpha = 0.99, indicating that interindividual differences in stacking remain stable over successive trials. Supplement 1 presents the changes in performance from pre- to posttest for males and females, showing that participants improved their stacking performance over the course of the study.

We fitted a linear mixed-effects model (using maximum likelihood estimation) to investigate the effects of spectators, performance prediction, gender, and age on performance in the stacking task. The model used the identification variable (“participant”) as a random effect. Its’ total explanatory power was large (conditional *R*^*2*^​ = 0.82), while the variance explained by fixed effects alone was small (marginal *R*^*2*^ = 0.15).

**Fig. 1:**
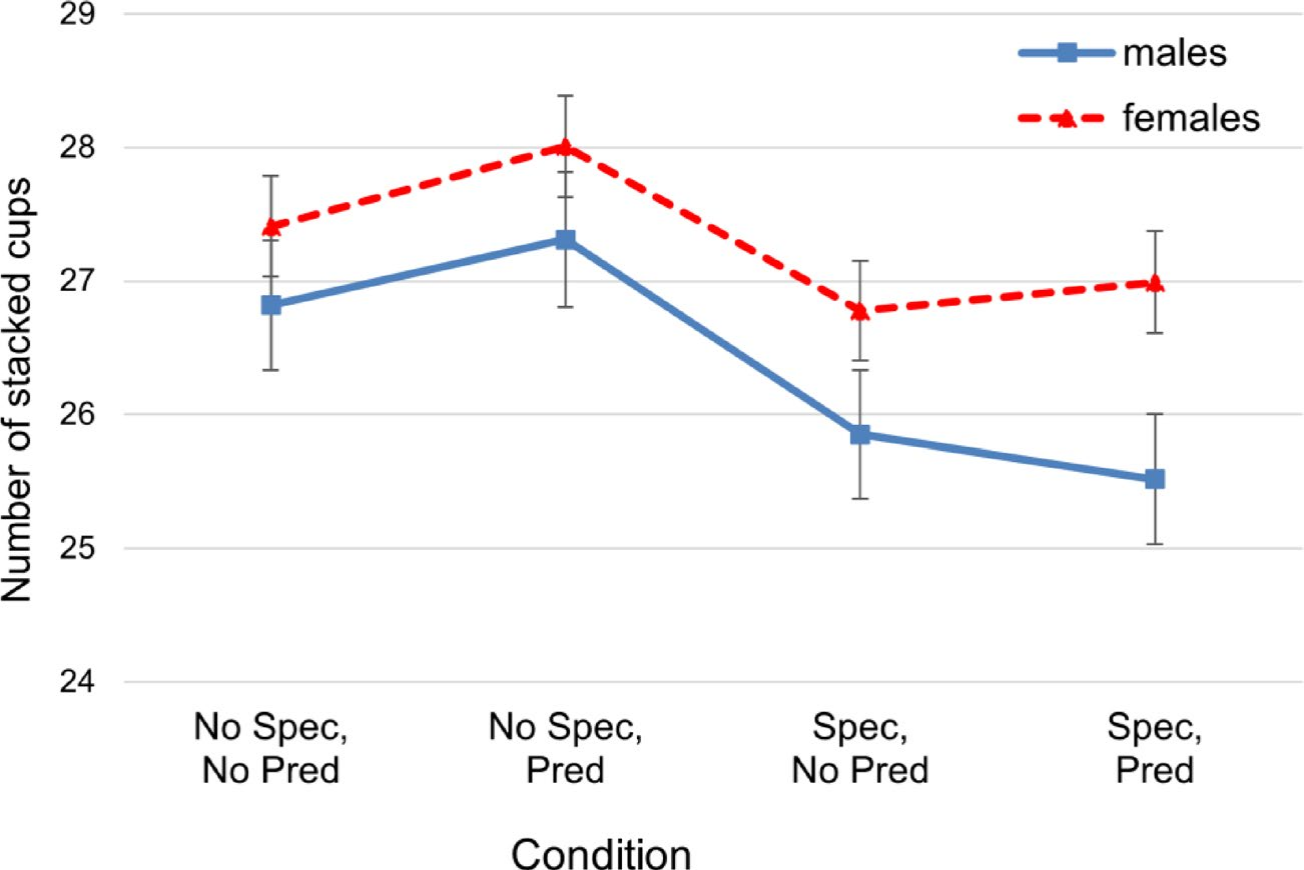
The Influence of Spectators and Performance Prediction on Stacking Performance of Males and Females. *Note*. Error bars = SE mean. Spec = spectators; Pred = performance prediction.

Results revealed a significant main effect of spectators (β = −0.97, 95% CI [−1.47, −0.47], *t*_(1019)_ = −3.79, *p* < .001), indicating that participants performed worse when being observed (Fig. 1). While the main effect of performance prediction showed a non-significant association with performance, gender had a significant effect, with females outperforming males (β = 1.39, 95% CI [0.31, 2.48], *t*_(1019)_ = 2.51, *p* =. 012) (Fig. 1). Age was significantly negatively associated with performance (β = −0.10, 95% CI [−0.13, −0.07], *t*_(1019)_ = −7.55, *p* < .001), suggesting a decline in the stacking performance with increasing age. A significant negative interaction between spectators and performance prediction (β = −0.83, 95% CI [−1.53, −0.12], *t*_(1019)_ = −2.28, *p* = .023) shows that the detrimental effect of spectators on performance was exacerbated when asking the participants to predict their performance. All other interactions failed to reach significance (for detailed values, see Table 1).

**Table 1:** Results of the linear mixed-effects model analyzing the effects on the stacking performance.

Effect	Estimate	SE	95% CI		*t*	*p*
			LL	UL		
Spectator	−0.97	0.26	−1.47	−0.47	−3.79	**< 0.001**
Performance prediction	0.49	0.26	−0.01	0.99	1.92	0.055
Gender (female)	1.39	0.56	0.31	2.48	2.51	**0.012**
Age	−0.10	0.01	−0.13	−0.07	−7.55	**< 0.001**
Spectator × Performance prediction	−0.83	0.36	−1.53	−0.12	−2.28	**0.023**
Spectator × Gender (female)	0.34	0.38	−0.40	1.07	0.90	0.370
Performance prediction × Gender (female)	0.11	0.38	−0.63	0.84	0.29	0.774
Spectator × Performance prediction × Gender (female)	0.44	0.53	−0.60	1.48	0.82	0.410

*Note*. Significant values are highlighted in bold for visual emphasis.

### Main hypothesis: selection margins by spectator condition and gender

SMS were calculated by subtracting the actual performance from the predicted score for each performance-prediction trial. Negative values represent an underestimation of one’s performance, and positive values represent an overestimation. Note that the paradigm should have motivated participants to predict slightly lower performances than their maximum-manageable task difficulty, since failing to reach the predicted performance led to the loss of all the points for the respective trial. The performance predictions seemed reasonable, and no participant entered highly unrealistic values, such as claiming that they could stack 100 cups in the upcoming trial. Furthermore, no participant provided unrealistically low estimates (e.g., 0 or 1 cups) in an attempt to secure their score for that trial.

**Fig. 2:**
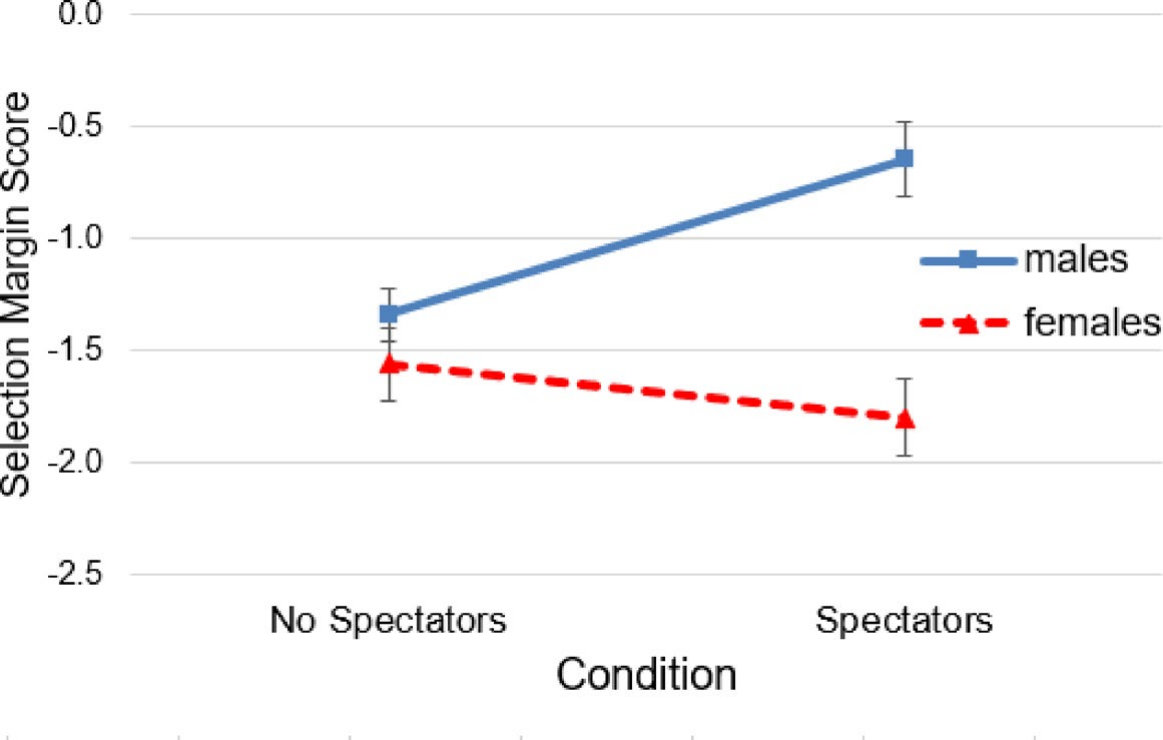
The Influence of Spectators on the Selection Margins Scores of Males and Females. *Note*. Error bars = SE mean.

Figure 2 presents the pattern of findings. A linear mixed-effects model was fitted to predict the selection margins scores based on spectators, gender, and age. The model was estimated using maximum likelihood and included the participant-identification variable as a random effect. The total explanatory power of the model was moderate (conditional *R*^*2*^​ = 0.35), and the part related to the fixed effects alone was small (marginal *R²* = 0.07).

A significant interaction was observed for spectators × gender (β = −1.07, 95% CI [−1.97, −0.17], *t*_(338)_ = −2.32, *p* = .021), indicating that when being watched, females showed significantly lower selection margins scores than males and therefore made more cautious performance predictions than in the co-action condition. In contrast, males showed considerably higher selection margins scores in front of spectators compared to the co-acting condition (see Table 2 for post hoc t-tests).

**Table 2:** Results of the post-hoc t-tests for the selection margins scores of males and Females.

Comparison	*t*	*df*	*p*
Males: No Spec vs. Spec	−3.34	328.47	0.001
Females: No Spec vs. Spec	1.00	313.13	0.318
Males vs. Females (Condition: No Spec)	−1.08	297.84	0.280
Males vs. Females (Condition: Spec)	4.77	336.2	< 0.001

*Note*. Rows one and two show the results of dependent t-tests, whereas the last two rows show the results of independent t-tests.

The main effects of gender, spectator and age as well as the interactions between spectator × age and gender × age were not significant. The three-way interaction of spectator × gender × age was also not significant (for detailed results, see Table 3).

**Table 3:** Results of the linear mixed-effects model analyzing the effects on the selection margin score.

Effect	Estimate	SE	95% CI		*t*	*p*
			LL	UL		
Spectator	0.37	0.32	−0.26	1.00	1.15	0.253
Gender	−0.74	0.39	−1.50	0.02	−1.90	0.058
Age	−0.01	0.01	−0.02	0.01	−0.52	0.604
Spectator × Gender	−1.07	0.46	−1.97	−0.17	−2.32	**0.021**
Spectator × Age	0.01	0.01	−0.01	0.03	1.20	0.230
Gender × Age	0.02	0.01	−0.01	0.04	1.60	0.110
Spectator × Gender × Age	0.01	0.01	−0.02	0.03	0.38	0.706

*Note*. Significant values are highlighted in bold for visual emphasis.

### Exploratory analyses: zero-point trials

**Fig. 3:**
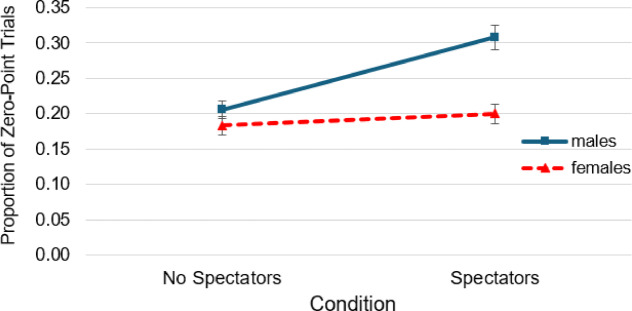
Proportion of Zero Point Trials for Males and Females. *Note*. Error bars = SE mean.

We conducted an exploratory additional analysis of the proportion of zero-point trials of males and females for the co-action and spectator condition. This analysis intended to capture individual instances of misjudgment on single trials that are not reflected in the mean selection margin scores averaged across participants. In total, 284 zero-point trials occurred out of 915 trials for men, and 158 zero-point trials occurred out of 790 trials for women. For the proportion of zero-point trials, the ANOVA with spectator (2; spectator, no spectator) as the within-subjects factor and gender (2; male, female) as the between-subjects factor revealed a significant main effect of gender, *F*(1, 339) = 17.82, *p* < .001, η²*p* = .05, indicating that males had a higher proportion of zero-point trials overall. A significant main effect of spectator was also found, *F*(1, 339) = 20.82, *p* < .001, η²*p* = .06, representing a higher proportion of zero-point trials in front of spectators. The analysis furthermore revealed a significant interaction of gender and spectator, *F*(1, 339) = 10.56, *p* = .001, η²*p* = .03, showing that the proportion of zero-point trials increased in the presence of spectators for males, whereas it remained unchanged for females (Fig. 3). The post-hoc *t*-test for the spectator condition showed that in front of spectators, males exhibited a significantly higher proportion of zero-point trials (*M* = 0.31, *SD* = 0.23) compared to females (*M* = 0.20, *SD* = 0.18), *t*_(339)_ **=** 4.82, *p* < .001.

### Exploratory analyses: trial-by-trial analyses of selection margins

**Fig. 4:**
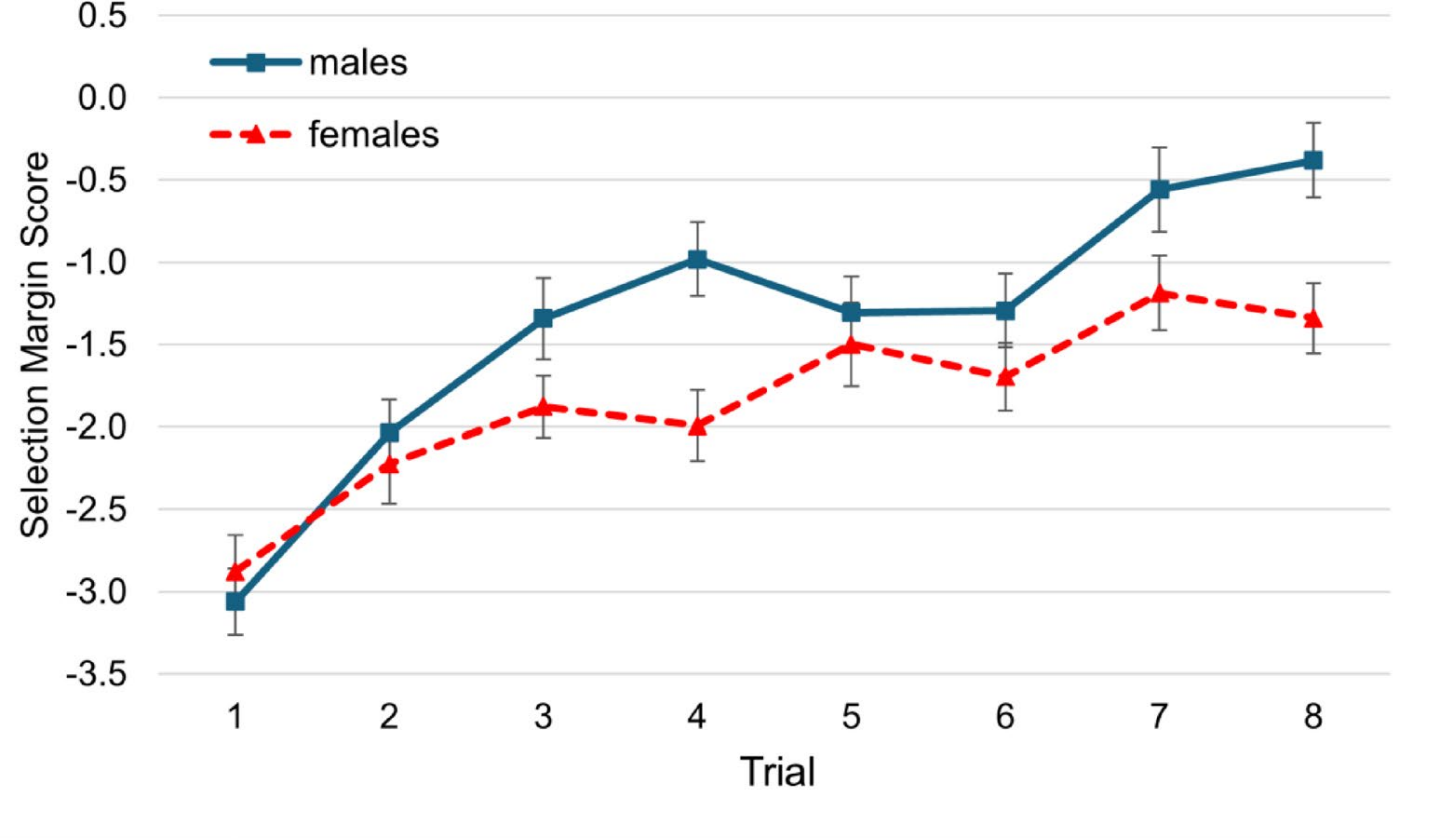
Selection Margin Scores of Males and Females over Trials for the Co-Action Condition. *Note*. Error bars = SE mean.

We conducted additional exploratory trial-by-trial analyses on the performance-prediction trials with and without spectators. We performed this exploratory analysis to gain further insight into how selection margin scores developed over the course of the co-action and spectator condition. Note that due to missing values, 301 participants were included in the analysis of the co-action condition. The co-action and spectator conditions were not performed in the same order for all participants, due to our counterbalancing scheme. For the co-acting condition, the ANOVA with trial (8) as within-subjects factor and gender (2; male, female) as between-subjects factor revealed a significant main effect of trial on SMS, *F*(7, 2093) = 9.89, *p* < .001, η²*p* = .03, which is mainly due to a linear tendency to become more progressive in one’s predictions (Fig. 4). The interaction of gender and trial failed to reach significance, *F*(7, 2093) = 0.87, *p* = .534, η²*p* = .00. There was a significant main effect of gender, *F*(1, 299) = 6.25, *p* = .013, η²*p* = .02 (Fig. 3), indicating that overall men showed more progressive selection margin scores.

**Fig. 5:**
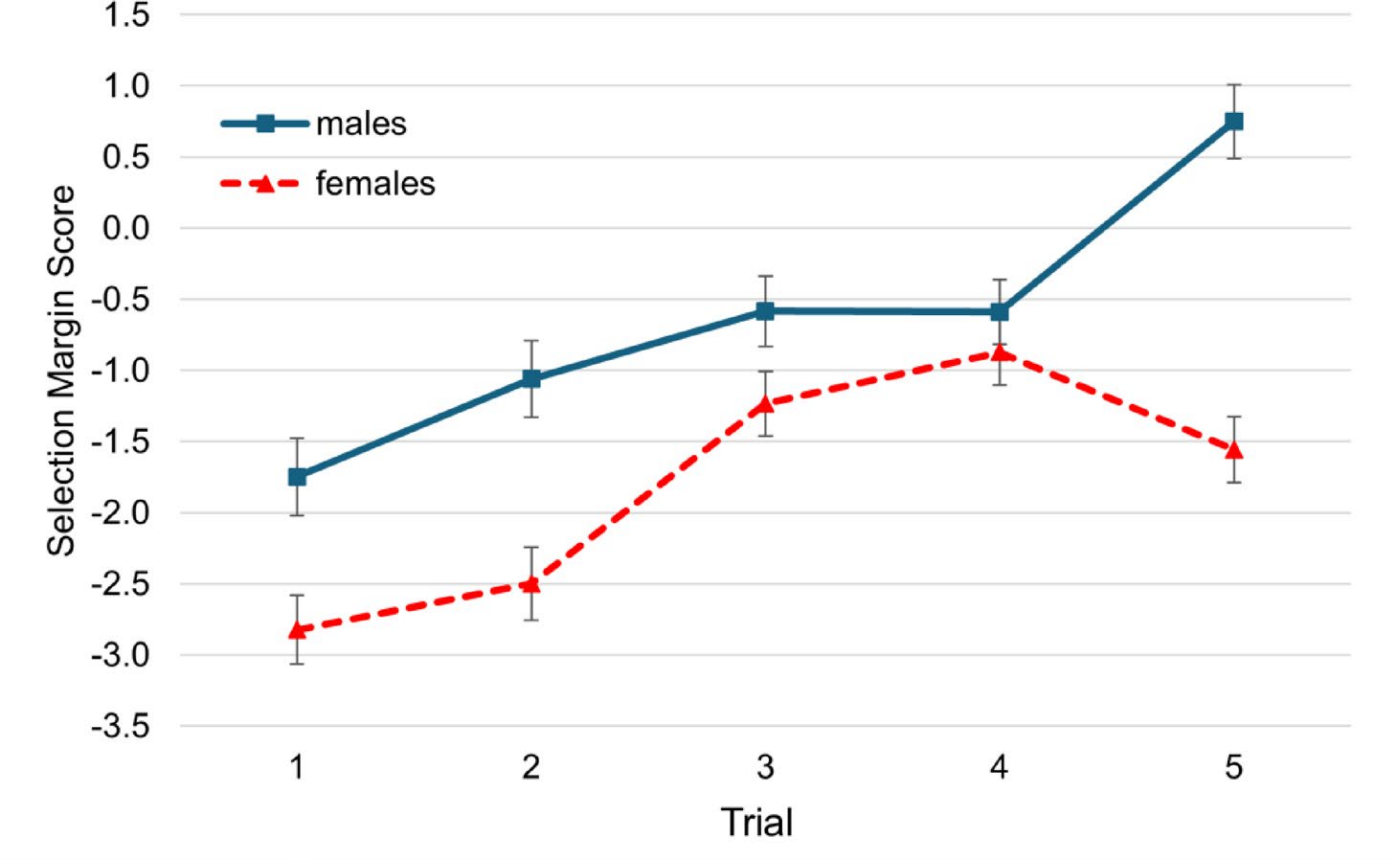
Selection Margin Scores of Males and Females over Trials for the Spectator Condition. *Note.* Error bars = SE mean.

When performing in front of spectators, there was a significant main effect of trial, *F*(4, 1356) = 10.49, *p* < .001, η²*p* = .03, and an interaction of trial and gender, *F*(4, 1356) = 2.60, *p* = .035, η²*p* = .01 The significant interaction indicates that the development of SMS across trials differed between males and females, with males showing a more progressive performance prediction pattern (the tendency to predict an increasing number of stacked cups). The main effect of gender also reached significance, *F*(1, 339) = 22.54, *p* < .001, η²*p* = .06 (Fig. 5). The post-hoc t-tests comparing the selection margin scores between males and females showed significant differences in trial 1, 2 and 5 of the spectator condition (see Table 4).

**Table 4:** Post-hoc t-tests for gender differences in the Trial-by-Trial analysis on selection margin scores in the spectator Condition.

Trial (spectator condition)	*t*	*df*	*p*
**1**	2.09	338.55	0.038
**2**	2.75	335.95	0.006
**3**	1.37	337.66	0.172
**4**	0.62	328.25	0.538
**5**	4.72	338.55	< 0.001

These additional analyses highlight the strategic decision-making of males and females over the course of the trials of performance-prediction with and without spectators. The risk-taking tendencies of males are particularly evident in the final trial in front of spectators, where males exhibit a positive selection margin score, reflecting an overestimation of their performance. In contrast, females show a shift to a more conservative selection margin, and thus leave a safety margin in their predictions.

## Discussion

The current study investigated the effects of spectators and gender on performance and self-assessment of one’s own performance in a cup-stacking task. The subjects performed the task in a co-acting condition, or while being watched by the other participants. In half of the trials, participants additionally predicted their performance for the upcoming trial. This paradigm encouraged participants to leave a safety margin in their predictions, as they lost all points for a specific trial if they failed to perform at least at the predicted level. At the same time, strongly underestimating one’s performance was also a suboptimal strategy, since participants consistently collected fewer points than they could have based on their ability.

We found that stacking performance is reduced when performing in front of an audience. This is consistent with the review by Strauss (2002) on spectator effects in motor tasks, predicting performance decrements for tasks that predominantly involve coordination^1^. In their recent systematic review and meta-analysis, van Meurs et al. (2024) suggest that coordination-based tasks are not consistently hindered by spectators^2^. According to the authors, object manipulation under precision pressure is inhibited, particularly when the task is not well-practiced, whereas object manipulation under time pressure is enhanced. Stacking as a motor task included precision and time pressure. Precision pressure is caused by the need to carefully place each cup in the correct position in the pyramid, because otherwise the pyramid would collapse. Time pressure is induced because participants had to construct and deconstruct their pyramids in a fixed time-window of 30 s for each trial. We also found significant gender differences in stacking, with females outperforming males, which corresponds to the literature on gender differences in fine-motor tasks^34–36^.

The interaction of gender and spectators on stacking performance was not significant, indicating similar performance deteriorations in the coordination-based motor task stacking in front of spectators for males and females. In contrast, recent work by Heinrich et al. (2021) on biathlon performance found that social facilitation effects in coordination tasks (rifle shooting) were gender-specific, with male biathletes showing performance deteriorations in the presence of an audience, whereas female biathletes exhibited performance improvements^29^. The current study also found age-related declines in stacking performances, corresponding to findings from a fine-motor precision task using a unimanual spiral-drawing task^37^.

Our spectator condition deviates from many classical studies of spectator effects, in which spectators were strangers who were merely present and were not involved in the task at all (for a review, see ^38^). In our study, participants were being watched by the other members of the testing group, and everyone knew that they would be asked to perform the task in front of the others at some point. Furthermore, in half of the trials in front of spectators, participants had to predict their own performance for the upcoming trial. The results of the present study demonstrate that incorporating these performance predictions further amplified the detrimental effect of spectators on stacking performance. This aligns with the evaluation approaches of social facilitation^3,39,40^, which posit that performance is affected not merely by the presence of others, but also by the anticipation of being evaluated. Requiring participants to predict their performance heightened their awareness of evaluation. This procedure increased the importance of a good performance, as failure would be immediately apparent to the spectators. Thus, choking under pressure may have also played a role in the exacerbated performance decrements^5^.

This was the first study to directly evaluate the effects of spectators on performance prediction in a motor task requiring precision and multi-joint coordination. Gender differences in self-assessment have been reported for different motor domains, revealing a tendency of males to overestimate their performance (e.g., in condition-based tasks such as long-distance running)^7,9,15–17^. When stacking simultaneously, males and females adjusted their performance predictions corresponding to their proficiency (leaving a safety margin). However, the presence of spectators led to gender differences in SMS, with males predicting higher performances than in the co-acting condition, whereas females remained rather cautious. The performance feedback after each trial allows for an adjustment of predictions from trial to trial. Persistently overestimating one’s own performance across multiple trials is a dysfunctional strategy, as it prevents effective adaptation to actual performance levels. Nevertheless, our results show that the presence of spectators encourages this maladaptive behavior in males. Being “pushed beyond their limits“ by the audience had detrimental consequences, as males lost significantly more points than females in unsuccessful trials, receiving zero points.

Males and females demonstrated an overall reasonable strategy in front of spectators, as shown in the trial-by-trial analysis. They became increasingly progressive in their SMS from trial to trial, with males being more progressive overall. However, the phenomenon of males overestimating their performance was particularly evident in the final stacking trial of the spectator condition.

The overestimations in the current study show a clear tendency for competition-seeking and risk-taking among males, possibly also influenced by ambitious goal setting. In contrast to males, females became even more cautious in the final trial in front of spectators, clearly underestimating their performance level, and therefore were able to secure points in the final trial as well. Recent research has shown that males and females respond differently to feedback on their motor performance. Kranzinger et al. (2024) analyzed how recreational skiers adjusted their self-assessed skiing ability after receiving objective feedback from a boot sensor system, which measured coordinative skiing quality (e.g., carving). The study found that after receiving feedback from the sensor system, women significantly aligned their self-evaluation with the objective performance score, whereas men showed little adjustment^41^. This is consistent with our findings, where males did not adjust their self-assessment of performance in front of spectators based on the received feedback. The results furthermore support existing evidence that males enter competition more often than females, even when no gender differences in performance are observed^38^. Niederle and Vesterlund (2007) argue that one main factor of this difference in tournament entry is that males are more overconfident than females^38^.

In the current study, the accuracy of performance predictions was not influenced by age. This stands in contrast to previous studies showing age differences in performance predictions for cognitive and motor tasks^19–22,42,43^. For an obstacle crossing task, overconfidence was shown in older adults^21^, whereas the extent of over- or underconfidence may also depend on the type of motor task. When only carrying a tray, older adults were more risk-tolerant in their performance predictions compared to stepping over a crossbar^22^. These findings align with the ‘posture-first’ principle, which has frequently been observed in older adults in demanding cognitive-motor dual-task situations^22^. Note that stacking in a seated position does not involve a risk of physical harm, and does not challenge posture.

In summary, spectators not only influenced performance but also self-assessment of performance in the stacking task of the current study. In front of an audience, overconfidence was increased in males but not females. Therefore, gender effects should be included in future social facilitation studies^29^.

### Limitations

For cognitive tasks, it was shown that the self-perception of one’s own performance differs depending on the task characteristics (e.g., mathematics, language skills)^44^. It is possible that self-assessment in motor tasks varies based on the specific motor abilities involved in the task (e.g., endurance, strength, or coordination)^45^. We focused exclusively on a specific motor task, speeded cup stacking. Participants appeared to be highly motivated to perform well, but the importance of such a task for real life may be limited. In studies investigating spectator effects, co-acting situations are often used as a control condition for spectator conditions. This was also the case in the present study. However, this approach can be questioned because the presence of co-performers may stillelicit social influences on performance. Furthermore, in the present study, the experimenter was also present in the room during the co-acting condition, which may have influenced participants’ behavior.

Our power analysis, conducted with GPower, does not account for the specific characteristics of the statistical model used. Therefore, the sample size estimation may not perfectly reflect the power for the analyses conducted. Future studies might address this limitation using simulation-based approaches to determine appropriate sample sizes more precisely.

Although the presence of spectators and experimenters likely minimized the possibility of intentional misreporting (cheating), we cannot completely exclude the potential for participants to inaccurately record their performance, particularly in the coaction condition where no permanent observation took place.

### Future directions

Future research should systematically examine different cognitive and motor tasks, which also differ in their perceived importance^46^. In addition, we did not systematically vary or control the gender composition of our testing groups. Since the gender composition of co-acting groups can influence individual performances^47^ or behavioral strategies^46^, these aspects should be controlled for in future research.

## Conclusion and implications

Overestimation and risk-taking in the stacking task primarily involved social punishment, as failure was observed by an audience. Males’ tendency toward overestimation and risk-taking may result in more severe consequences (e.g., physical harm, or financial losses in gambling), but it may sometimes also be advantageous (e.g., pushing oneself to maximum performance, convincing others in job interviews).

## Supplementary Information

Below is the link to the electronic supplementary material.

Supplementary Material 1

## Data Availability

Data and the analysis code can be accessed here: https://osf.io/bvc3s/?view_only=1a835e6a602b49cc9c3c2d59d15327a7.
