# Supplementary material for: Spectators lead to overconfidence and risk-taking in males in a motor task
Source: Sci Rep. 2025 Sep 12;15:32449. doi: 10.1038/s41598-025-18048-0 (PMC12432250; doi:10.1038/s41598-025-18048-0)
Supplement: Supplementary file 1 — Supplementary Material 1 [file 41598_2025_18048_MOESM1_ESM.docx]

**Changes in Stacking Performance from Pre- to Posttest**

Participants performed 10 stacking trials in the pretest phase of the study, and 8 stacking trials in the posttest phase, under co-acting conditions. Figure S1 present performance changes from pre- to posttest for males and females separately.

**Figure S1**

Stacking Performance Changes from Pre- to Posttest in Males and Females


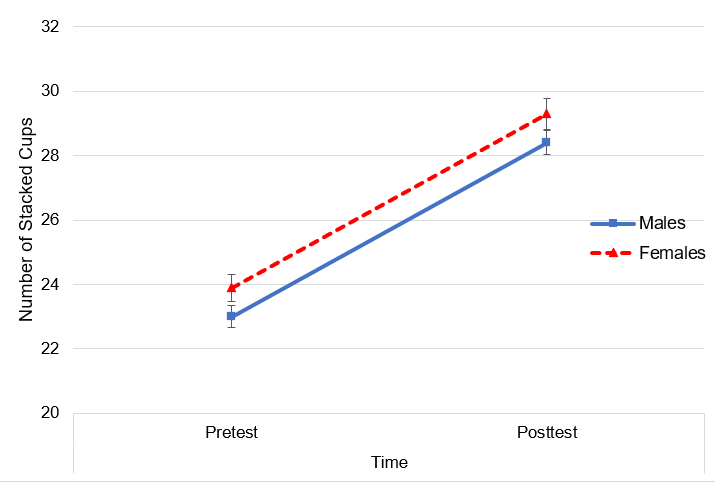


*Note:* Error bars = SE means

We conducted a mixed-design ANOVA with time (2: pre- versus posttest) as repeated-measures factor and gender (2: males versus females) as between-subjects factor. The ANOVA revealed a significant main effect of time, *F*(1, 673) = 179.82, *p* < .001, *η²p* = .21, and a main effect of gender, *F*(1, 673) = 4.35, *p* = .037, *η²p* = .006, and no interaction of time and gender, *F*(1, 673) = .005, *p* = .944, *η²p* < .001. Participants improved their stacking performances over the course of the study, indicating that fatigue or loss of motivation did not play a major role for the current data set.
